# Molecular validation of carnivore scat surveys: Effects of climate, scat age, and observer experience on identification success

**DOI:** 10.1371/journal.pone.0343095

**Published:** 2026-02-23

**Authors:** Francisco Palomares, Jacinto Román, Javier Calzada, Juan Carlos Rivilla, Irene Quintanilla

**Affiliations:** 1 Department of Conservation Biology and Climate Change, Estación Biológica de Doñana-CSIC, Sevilla, Spain,; 2 Departamento de Ciencias Integradas y Centro de Estudios Avanzados en Física, Matemáticas y Computación, Facultad de Ciencias Experimentales, Universidad de Huelva, Huelva, Spain; Universitat Autonoma de Barcelona, SPAIN

## Abstract

Non-invasive genetic sampling has become an essential tool for monitoring carnivores; however, the success of molecular identifications from scats varies widely across taxa, environments, and observers. Field-based assignments are also prone to misclassification, particularly when species are sympatric and produce similar scats. Understanding the determinants of molecular success and the concordance between field and genetic identifications is therefore critical for designing reliable surveys. We analysed 2,073 carnivore scats collected across five protected areas in Spain. A binomial GLMM showed that scat age, climatic conditions, observer identity, and year significantly influenced the probability of successful genetic identification. Fresh scats had nearly double the odds of yielding a valid genetic result compared with medium-aged scats, whereas higher precipitation and temperatures reduced success. Observer differences were also evident, and interannual variation suggested the presence of additional environmental effects. Of the 1,835 scats successfully identified, the overall match rate between field and molecular assignments was 75.9% (Cohen’s κ = 0.68), increasing to 80.0% (κ = 0.74) when genus-rank identifications (*Canis* sp., *Felis* sp.) were considered correct. High-confidence field identifications achieved>90% agreement, but congeneric species, such as *Martes foina* and *M. martes*, were frequently misclassified. Our findings demonstrate that the interaction of environmental and human factors influences the success of identification. Field-based identifications, although often reliable, can lead to systematic biases in species-rich assemblages. We recommend incorporating molecular validation into carnivore surveys whenever species-level precision is required, especially for rare or threatened taxa.

## Introduction

Non-invasive genetic sampling has become a cornerstone in wildlife ecology and conservation, particularly for elusive carnivores where direct observations or captures are logistically challenging and ethically sensitive. Scat collection in the field provides an accessible source of DNA that can be used to identify species, individuals, sex, and to obtain valuable information on presence, population size, interspecific interactions, and foraging ecology, among others [1–3]. Nevertheless, the reliability of these samples is influenced by multiple factors. DNA quality often declines due to environmental exposure, degradation over time, and the presence of inhibitory substances in faecal matter, resulting in variable success rates across studies and species [2,4,5]. Understanding the determinants of molecular success is therefore crucial for designing effective sampling protocols and maximizing the information obtained from field collections.

In parallel, field assignment of scats to species has been a common practice in ecological surveys [e.g. 6,7]. Experienced observers often rely on morphology, odor, and associated field signs such as prints, specific deposition characteristics, and other contextual evidence to classify scats, yet such identifications are prone to error when species are sympatric and produce similar deposits [6–10]. Misclassification not only reduces the reliability of ecological inferences but also complicates conservation decisions when species of concern are involved. Comparative studies have shown that low-confidence field assignments may bias ecological conclusions relative to high-confidence genetic or chemical identifications [7]. In this context, Lonsinger et al. [6] demonstrated that scat identification based on morphometric measurements alone is not fully reliable for closely related sympatric species. Despite the widespread use of both field and molecular approaches, few studies have systematically evaluated their concordance across diverse carnivore communities and environmental conditions.

In this study, we used a large dataset of carnivore scats collected across five protected areas in Spain, spanning broad climatic gradients and supporting contrasting carnivore assemblages. Our objectives were twofold: (1) to evaluate how local weather conditions, scat age, and observer influence the probability of obtaining molecular species identification from scats, and (2) to quantify the degree of concordance between field-based and genetic species assignments. By addressing these questions, we aim to provide empirical guidance on the reliability of scat surveys and the circumstances under which molecular validation is most critical.

### Study area

**Fig 1:**
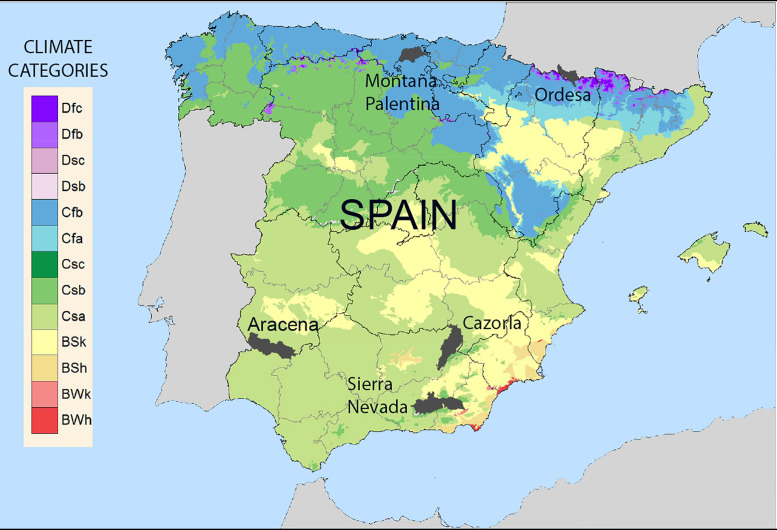
Study areas in the Iberian Peninsula where wild carnivore scats were collected to evaluate the reliability of scat identifications. Black polygons show the limits of the five protected areas surveyed: Aracena, Cazorla, Montaña Palentina, Sierra Nevada, and Ordesa. The background map shows the main climatic categories of the Iberian Peninsula according to the Köppen–Geiger classification (obtained from [11]), illustrating the climatic context in which the study areas are located. A detailed description of the climatic categories affecting each study area is provided in the Supporting Information.

The study areas in Spain were the Natural Park of the Sierra de Aracena and Picos de Aroche (thereafter Aracena), the Natural Park of the Sierras de Cazorla, Segura and Las Villas (thereafter Cazorla), Sierra Nevada Natural and National Park (thereafter Sierra Nevada), Montaña Palentina Natural Park (thereafter Montaña Palentina), and Ordesa and Monte Perdido National Park (thereafter Ordesa), and the close surrounding areas to them to increase heterogeneity and anthropization of the sampled areas (Fig 1).

The five study areas span a broad latitudinal and ecological gradient across the Iberian Peninsula, including Mediterranean mountain systems in the south (Aracena, Cazorla, and Sierra Nevada) and Eurosiberian-influenced ranges in the north (Montaña Palentina and Ordesa). Southern areas are generally warmer and drier (S1 File; Fig 1), with higher levels of anthropogenic land use (e.g., dehesas, olive groves, pine plantations). In contrast, northern areas are cooler and wetter, dominated by deciduous and coniferous forests, alpine meadows, and extensive pastures (S1 File; Fig 1).

All sites support diverse wild carnivore communities. Shared mesocarnivores include red fox (*Vulpes vulpes*), European badger (*Meles meles*), stone marten (*Martes foina*), European wildcat (*Felis silvestris*), common genet (*Genetta genetta*), polecat (*Mustela putorius*), and weasel (*Mustela nivalis*). Additional species occur regionally, such as Egyptian mongoose (*Herpestes ichneumon*) in Aracena, pine marten (*Martes martes)* and stoat *(Mustela erminea)* in the northern areas, and Eurasian otter (*Lutra lutra*) in all areas except Sierra Nevada. Large carnivores, such as the wolf (*Canis lupus*) and the brown bear (*Ursus arctos*), are restricted to northern systems. This variation in climate, land use, and community composition provides a heterogeneous framework for evaluating factors influencing the success of molecular identification in carnivore scats.

## Methods

### Field sampling

Wild carnivore scats were collected during systematic surveys in winter (January, February, or March) and summer (June or July) 2022/2023 across Aracena, Cazorla, and Montaña Palentina, as well as in winter (January) and summer (July) 2023 in Sierra Nevada, and in summer (September) 2023 in Ordesa. Normally, we spend three days sampling in each area and season, but sometimes we add an extra day to get more samples for some species. We estimated that we walked between 81 and 148 km per area and season (authors, unpub. Information). We attempted to collect scat from all previously enumerated species, except those of brown bears and Eurasian otter, as the spatial scale of their occurrence fell outside the objectives of this study.

Survey routes were designed from detailed maps to maximize landscape heterogeneity and were conducted on foot along paths, streams, near burrows, and in dense vegetation patches. Each scat was provisionally assigned to a putative carnivore species based on morphology, appearance, odor, and associated contextual field signs. Sampling was primarily conducted by four observers (FP, JR, JC and JCR) with over 30 years of experience working with carnivores.

For each scat, geographic coordinates were recorded with the CyberTracker application, generating a unique sample code. We also assigned a qualitative confidence level to field-based species identification (high, medium, or low following [12]). High indicated strong certainty in the field identification, medium reflected some uncertainty while still favouring the assigned species, and low denoted cases where distinguishing among two or more carnivore species in the field was challenging.

For molecular analyses, we hydrated the surface of the scats with a preservative solution [13], and a sample was obtained by rubbing a swab moistened with the same buffer on the surface of the excrement. The swab was preserved submerged in a labelled Eppendorf tube with the same buffer. The scat itself was stored in a paper bag with silica gel, dried at 60 °C in a fume hood, and subsequently archived in sealed bags under dry, dark conditions until analysis. All samples were handled with disposable gloves to avoid contamination.

### Molecular identification of scats

DNA extractions were carried out under sterile conditions at the Molecular Ecology Laboratory (LEM, EBD-CSIC) following standard protocols for degraded or low-quantity samples [14,15], with a slightly adaptation of the GuSCN/silica protocol. Instead of extracting samples in individual Eppendorf tubes, we used glass-fiber filter plates (MultiScreen 96-well High Volume Filter Plate, 1.2 µm pore size).

Initially, mitochondrial cytochrome b primers [16] were used to identify carnivore species. When amplification failed, a larger amount of DNA extract was used and/or a new extraction was performed from the same scat. In addition, samples that showed poor amplification or ambiguous results were subjected to Sanger sequencing of the ATP6 marker to confirm species identity. Samples that initially failed were typically re-tested two to three times. However, this method could not discriminate between wildcats and domestic cats or between wolves and dogs. Therefore, a panel of 96 nuclear SNP markers was applied for wildcat identification [17], and a fragment of the mitochondrial control region was sequenced in *Canis* samples to distinguish wolves from dogs [18].

Detailed field and laboratory procedures for all genetic protocols are provided in the dataset repository [19].

### Data analyses

Before data analysis, we applied some filtering criteria to the data presented in the public repository [19]. Domestic cat and dog samples were not considered, as the focus was restricted to wild carnivores. Genet and badger scats were not included, as these were consistently identified with certainty in the field due to their specific places of defecation, which make them unmistakable [20]. Therefore, molecular techniques were not used to confirm the species. To ensure consistency in field identifications, we only retained observations made by the four core observers (FP, JR, JC and JCR); a few records from other collaborators were excluded. When students or assistants accompanied senior observers, only the senior observer was recorded. In cases where two or more senior observers surveyed together, the observer identity was coded as “Group.” Finally, for some analyses, molecular results that identified scats only to the genus rank (*Canis* sp., *Felis* sp.) were recoded as *Canis lupus* and *Felis silvestris*, respectively, to reflect assumed concordance.

We quantified overall agreement between field and molecular identifications as the proportion of correctly classified scats (sum of diagonal elements/total number of cases) and calculated Cohen’s κ to assess concordance beyond chance.

To examine the effect of climate, observer, and age on the probability of scat successful identification by molecular techniques, we modelled the probability that a scat sample yielded a successful genetic identification (response, 0/1) with a binomial GLMM (logit link) using the lme4 package in R (version 1.1–35.4 [21];). Fixed effects were: scat age category (*Scat_age*; reference = “Medium”), centred precipitation (Precip_c), centred mean temperature (Mean_Temp_c), observer identity (*Observer_1*), and year (*Year*; reference = 2022). “Area” (5 levels) was included as a random intercept to account for spatial clustering. We previously tested (i) the interaction *Scat_age × Precip_c* and (ii) sampling (winter 22, summer 22, winter 23, and summer 23) as covariates, but these terms were not significant and increased model complexity, so they were excluded from the final model. We also considered four climatic variables (obtained from the Agency for Meteorological Services and Atmospheric Research of Spain (AEMET; https://www.aemet.es): mean temperature (°C), total precipitation, the number of days with rainfall ≥ 1 mm, and average relative humidity (%) for the one month preceding and including the sampling days. The mean temperature was highly correlated with relative humidity (r = −0.718), and precipitation was associated with the number of days with rainfall ≥ 1 mm (r = 0.812) and relative humidity (r = 0.964). Therefore, we included only mean temperature and total precipitation in the final model. Continuous climatic predictors were mean-centred to aid interpretation. Model fit was assessed with DHARMa (version 0.4.6 [22]) residual diagnostics, intraclass correlation coefficient (ICC), and marginal/conditional R². Odds ratios (OR) and 95% Wald confidence intervals were calculated by exponentiating the fixed-effect estimates.

To compare field and molecular identifications, we only considered the scats that molecular techniques could identify. We evaluated the factors influencing the probability of correct identification of field-collected scats using a generalized linear mixed-effects model with a binomial (logit) link function. Before analysis, field assignments of *Felis* sp. and *Canis* sp. were recoded to *Felis silvestris* and *Canis lupus*, respectively, to reflect assumed concordance. Our response variable was a binary indicator of concordance between field and molecular species assignments. Fixed effects included observer identity, scat age (fresh, medium, old), field‐identification certainty (high, medium, low), sampling session (Win_22, Sum_22, Win_23, Sum_23), and year (2022, 2023); area was entered as a random intercept to account for spatial clustering across the five study regions. This approach allowed us to test whether (1) observer experience, scat age, or certainty influence field identification success, (2) success rates have changed across sampling sessions or between years, and (3) there remains residual variation among areas. Predictors were dummy-coded or factored with reference levels (e.g., Win_22 for sampling, with high as the reference level for certainty). Model fit was assessed using AIC and marginal/conditional R², and residual diagnostics were performed with the DHARMa package (Version 0.4.6 [22]) to check for overdispersion and outliers. Odds ratios and 95% Wald confidence intervals were computed from the fixed‐effect estimates, and population‐level effect predictions for each categorical variable were visualized with bias‐corrected marginal plots generated by the ggeffects package (version 2.3.0 [23]).

## Results

### Factors influencing the molecular identification of scats

Molecular identification was performed on a total of 2073 scats collected by the four central observers involved in the sampling. Out of 2073 scats, 11.5% (238) could not be identified by molecular analyses, while the remaining ones were assigned to different species (see below).

**Fig 2:**
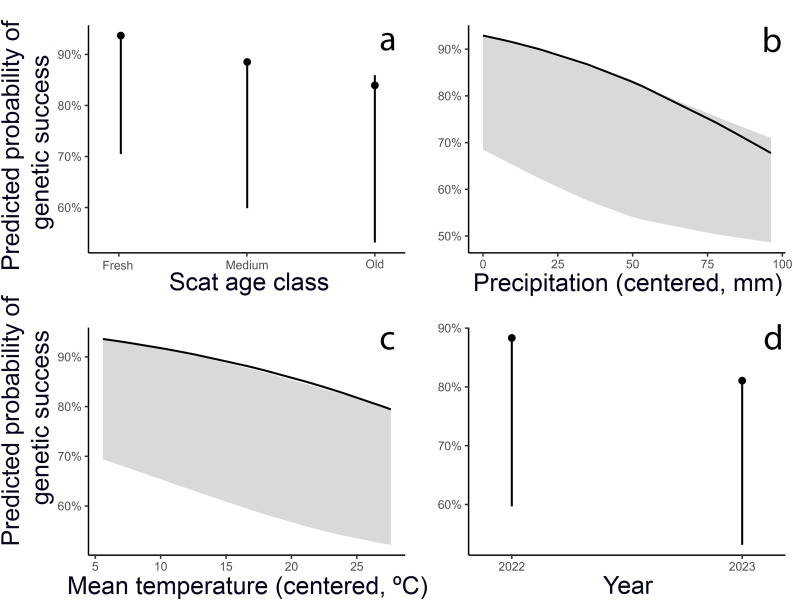
Combined panel of marginal effects: (a) Scat age, (b) Precipitation, (c) Temperature, and (d) Year. Each panel shows predicted probabilities with 95% CIs, derived from the GLMM while integrating over remaining predictors.

**Fig 3:**
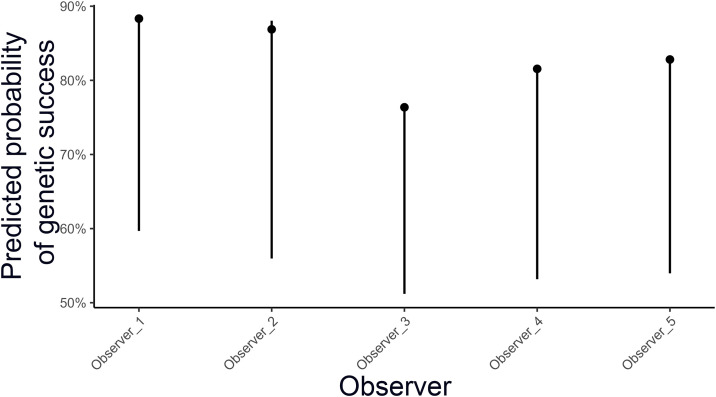
Predicted probabilities (±95% CI) for each observer (Observer_2, Observer_3, Observer_4, Observer_5) on genetic success. Estimates are adjusted for all other covariates.

The binomial GLMM model (AIC = 1266.2) showed a significant effect of scat age, precipitation, temperature, observer, and year (Table 1; Figs 2 and 3). Samples classified as “Fresh” had nearly double the odds of successful identification compared with “Medium” (OR = 1.98, 95% CI 1.43–2.74, *p* < 0.001). “Old” samples did not differ from “Medium” ones (OR = 0.68, 0.34–1.38, *p* = 0.29).

**Table 1:** Fixed‐effect estimates from the binomial GLMM predicting the probability of genetic identification of scats in seven carnivore species from the Iberian Peninsula. Odds ratios (OR), 95% Wald confidence intervals (CI), and p‑values are shown. The reference levels are Scat Age = Medium, Observer = Observer 1, and Year = 2022. Significant predictors (*p* < 0.05) include fresh scat age, precipitation, mean temperature, Observer 3, Observer 4, Observer 5, and Year 2023.

Predictor	OR	95% CI	p-value
Intercept	18.03	7.18–45.24	<0.001
Scat Age: Fresh	1.98	1.43–2.74	<0.001
Scat Age: Old	0.68	0.34–1.38	0.290
Precip_c (mm)	0.98	0.97–0.99	<0.001
Mean_T_c (°C)	0.94	0.91–0.97	<0.001
Observer: Obser. 2	0.87	0.44–1.72	0.692
Observer: Obser. 3	0.41	0.27–0.63	<0.001
Observer: Obser. 4	0.57	0.36–0.92	0.020
Observer: Obser. 5	0.63	0.40–0.98	0.042
Year 2023	0.55	0.35–0.87	0.011

Both climatic variables were negatively associated with success: each additional mm of precipitation (relative to the mean) slightly reduced the odds (OR = 0.98, 0.97–0.99, *p* < 0.0001), and higher mean temperatures also decreased success (OR = 0.94 per °C, 0.91–0.97, *p* < 0.0001).

Year also had a significant effect, with 2023 showing lower odds of identification than 2022 (OR = 0.55, 0.35–0.87, *p* = 0.011). Observer differences were evident; compared with the reference observer, Observer_3 (OR = 0.41, 0.27–0.63), Observer_4 (OR = 0.57, 0.36–0.92), and Observer_5 (OR = 0.63, 0.40–0.98) had significantly lower success rates, whereas Observer_2 did not differ (Fig 3).

The random intercept for area had variance = 0.85 (SD = 0.92), yielding an adjusted ICC of 0.205, indicating that about 20% of the residual variance (on the logit scale) is attributable to differences among areas. The model’s marginal R² was 0.162 (variance explained by fixed effects alone), and the conditional R² was 0.334 (fixed + random effects). DHARMa diagnostics indicated no substantial dispersion, outliers, or deviation from uniform residuals.

### Comparison between field and molecular identifications

**Fig 4:**
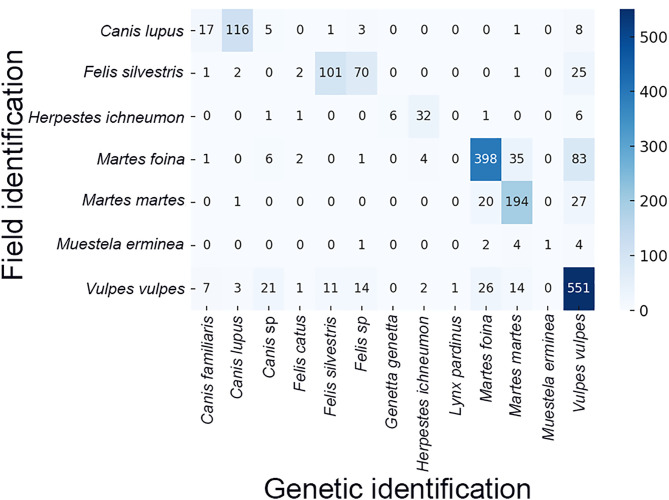
Heatmap of concordance between field-based and genetic species identifications. Each cell shows the count of scats assigned to a given species in the field (rows) and by genetic analysis (columns). Diagonal cells (shaded in darker blue) indicate matches between field and genetic identifications. Off-diagonal cells reveal misclassifications or broader-level genetic identifications (e.g., *Felis* sp. for field-identified *F. silvestris*). Color intensity corresponds to the number of samples in each cell.

After removing the scats that genetic analyses could not identify, we retained 1,835 samples. The global math rate between field and genetic identification was 75.9% with a Cohen’s K of 0.68, indicating a substantial agreement (Fig 4). The only species with a low coincidence rate was *Mustela erminea*, where only one from 12 putative scats was assigned to this species (Fig 4). As a rule, the more common confusions between field and genetic assignment were with taxonomically close species, even so often scats from *V. vulpes* may often be assigned to *F. silvestris*, *M. foina* and *M. martes* (Fig 4).

If we considered the scats identified as *Felis* sp by genetic analyses and *Felis silvestris* in the field, and those of *Canis* sp by genetic analyses and *Canis lupus* in the field as correct, the match rate increased to 80.0%, and the Cohen’s κ to 0.74.

The cases of *Martes foina* and *Martes martes* also deserve further consideration (Table 2). The overall rate of coincidence between field and genetic identification for *Martes foina* was 75.1%. Still, this figure increased to 83.2% when considering areas with the only presence of *Martes foina*, and decreased to 41.2% in areas where *Martes foina* and *Martes martes* coexisted. However, the percentage of coincidence of *Martes martes* in areas of coexistence of both species was 80.2%.

**Table 2:** Concordance rates between field-based and genetic identifications for *Martes foina* and *Martes martes* scats in different scenarios.

Area	Field species	Genetic species	
		*M. foina*	*M. martes*
**All areas**	*M. foina*	398 (75.1%) n = 530	–
**Areas with only *M. foina***	*M. foina*	356 (83.2%) n = 428	–
**Areas with both species**	*M. foina*	42 (41.2%) n = 102	–
	*M. martes*	–	194 (80.2%) n = 242

**Fig 5:**
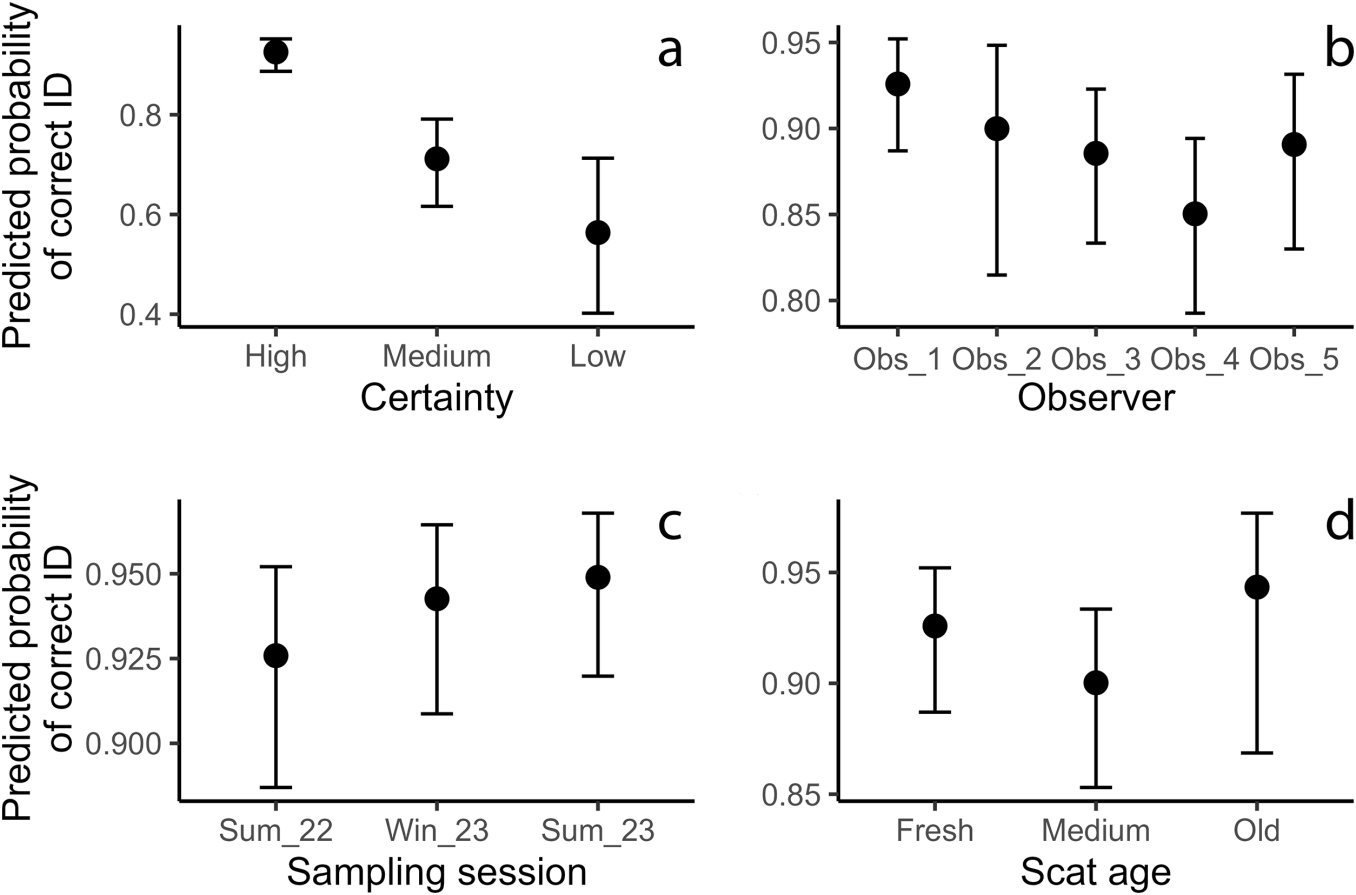
Predicted probability of correct field identification across key categorical predictors. Panels show marginal effects (point estimates ± 95% confidence intervals) from the final GLMM with area as a random effect. (a) Field‐identification certainty. (b) Observer identity. (c) Sampling session. (d) Scat age. All probabilities are back‐transformed on the response scale from the logit link. Error bars indicate 95% Wald confidence intervals.

Our final mixed‐effects model revealed that field identification success was predominantly driven by field‐identification certainty and observer identity, with negligible spatial effects (Table 3, Fig 5). Scats collected with “medium” certainty had 78% lower odds of correct field identification compared to those with “high” certainty (OR = 0.22, 95% CI 0.16–0.30, p < 0.001), and “low” certainty scats showed a 90% reduction (OR = 0.10, 95% CI 0.06–0.19, p < 0.001). Relative to the reference observer, Observer_3 and Observer_4 exhibited significantly lower agreement rates (OR = 0.62, 95% CI 0.41–0.95, p = 0.026; OR = 0.46, 95% CI 0.30–0.69, p < 0.001, respectively). Scat age had a modest effect: “medium” aged scats were less likely to yield correct field IDs than “fresh” (OR = 0.72, 95% CI 0.53–0.98, p = 0.038), whereas “old” scats did not differ significantly from fresh. Sampling session and year exerted a minor influence. Only Summer 2023 showed a slight increase in agreement (OR = 1.49, 95% CI 1.01–2.20, p = 0.045). Random‐effect variance for area was very low (σ = 0.185), and the adjusted ICC was 0.01, indicating minimal between‐area heterogeneity. Marginal R² for fixed effects was 0.19, and conditional R² including area was 0.20.

**Table 3:** Odds ratios (OR) and 95% Wald confidence intervals (CI) for predictors of correct field identification of scats. Estimates are derived from a generalized linear mixed-effects model (logit link) with area as a random intercept. The reference levels are Observer_1, Fresh scat age, High certainty, and Win_22 sampling session. P-values are from Wald z-tests. The effect of the area was minimal as σ²Area = 0.034 (σ = 0.185), adjusted ICC = 0.01.

Predictor	Odds Ratio	95% CI low	95% CI high	p‑value
**(Intercept)**	12.487	7.839	19.891	<0.001
**ObserverObserver_2**	0.720	0.377	1.375	0.319
**ObserverObserver_3**	0.619	0.406	0.945	0.026
**ObserverObserver_4**	0.455	0.301	0.688	<0.001
**ObserverObserver_5**	0.653	0.405	1.051	0.079
**Scat_ageMedium**	0.723	0.532	0.982	0.038
**Scat_ageOld**	1.334	0.559	3.187	0.516
**CertaintyMedium**	0.198	0.144	0.271	<0.001
**CertaintyLow**	0.103	0.056	0.193	<0.001
**SamplingWin_23**	1.316	0.883	1.959	0.177
**SamplingSum_23**	1.489	1.009	2.196	0.045

If we only considered the scats assigned as high certainty during field collection, the match rate increased to 90.4% and the Cohen’s κ was 0.867.

## Discussion

Our study examined two central questions: which factors influence the success of molecular identification of carnivore scats, and to what extent field-based assignments agree with genetic identifications. Together, these analyses provide insights into both the methodological reliability of non-invasive sampling and the value of molecular validation in carnivore ecology.

By combining large sample sizes, multiple carnivore species, and contrasting climatic regions, our study directly addresses two major gaps in the literature: the lack of broad-scale evaluations of molecular identification success under heterogeneous environmental conditions, and the limited empirical assessment of field–genetic concordance across species-rich carnivore assemblages.

Our dataset is one of the most extensive used to date in evaluating the reliability of non-invasive genetic sampling in carnivores. In contrast to most previous studies, which were restricted to a single species or a single region (e.g. [5,6,8–10]), our sampling encompassed more than two thousand scats across five protected areas that differ markedly in climate, habitat composition, and carnivore assemblages. As highlighted by Beja-Pereira et al. [2], most non-invasive genetic studies have involved relatively modest sample sizes and limited environmental variation, leaving open questions about the generality of their conclusions. By covering environments ranging from humid Atlantic to arid Mediterranean systems, our dataset provides a broader and more robust evaluation of the factors influencing molecular performance and field–genetic concordance than has been achieved in most previous assessments. This broader spatial and taxonomic scope allows us to assess the generality of patterns previously reported in more restricted settings, and to identify sources of variation that could not be detected in single-species or single-region studies.

We found that scat age, climatic conditions, observer identity, and year significantly affected the probability of successful genetic identification. Fresh scats were more likely to yield DNA of sufficient quality, confirming that degradation over time is a significant constraint [2,5,12]. The adverse effects of precipitation and higher temperatures also highlight the role of environmental exposure, which accelerates DNA breakdown and promotes microbial activity (also see [4] for ungulate samples). These findings stress the importance of timely collection and adequate storage protocols across environmental contexts, given that DNA degradation can occur rapidly under both hot-dry conditions and humid environments.

Differences among observers were also marked, despite all of them being experienced. Such variation may reflect subtle differences in scat handling or in the microhabitats chosen for surveys, reinforcing previous evidence that field personnel contribute to heterogeneity in molecular success [3]. The decline observed between 2022 and 2023 further suggests that unmeasured seasonal or interannual factors (possibly linked to weather conditions) or subtle, unidentified differences in laboratory analytical protocols can influence DNA preservation at a landscape scale. The only change we made to the laboratory protocol for analysing the faecal samples was that, in November 2022, the incubator thermostat used for sample digestions broke. Thus, the digestion of samples collected after that date (i.e., winter and summer 2023) was performed at room temperature instead of 37°C. The enzyme used was effective in the range of 20–65°C, so we do not think this should be a significant factor. Overall, these results underscore that molecular success is not uniform but shaped by interacting biological, environmental, and human factors, and that these should be explicitly considered when designing non-invasive surveys.

Agreement between field and genetic identifications was generally high (κ ≈ 0.7–0.74). The main misclassifications occurred among closely related or morphologically similar species, such as wildcats and domestic cats, or between stone and pine martens. This pattern mirrors previous reports of frequent errors in field-based scat assignments [6,8,10]. Importantly, field identifications made with high certainty reached an agreement of over 90%, suggesting that experienced observers can often be reliable when diagnostic features are clear. Nonetheless, even small error rates can bias ecological inferences, particularly when dealing with rare or threatened species [7,9,12].

Our results also indicate that the coexistence of morphologically similar species amplifies error rates. For instance, martens were frequently misclassified in areas of sympatry, consistent with the notion that sympatric assemblages challenge field assignments and reinforce the need for genetic validation. This has practical consequences: conservation assessments or diet studies that rely solely on field identifications risk over- or under-estimating the presence of particular carnivore species, leading to misleading conclusions [7].

## Implications for ecological and conservation research

Together, these findings highlight the trade-offs between field efficiency and molecular accuracy. Field assignments remain indispensable for conducting rapid surveys and broad-scale monitoring, especially in resource-limited settings. However, our results confirm that genetic validation substantially increases reliability, particularly under challenging conditions (old scats, adverse climate, species-rich assemblages). For ecological applications where species-level precision is essential, such as monitoring rare carnivores or quantifying interspecific interactions, molecular confirmation should be considered a standard practice.

Future studies should aim to optimize both field and laboratory protocols, incorporating training for observers, prioritizing the collection of fresh scats, and developing predictive models that account for environmental and observer-related sources of variation. In particular, integrating fine-scale microclimatic data, experimental comparisons of preservation methods, and cross-validation across independent observer teams would further improve the robustness of non-invasive genetic surveys.

## Supporting information

S1 File Temperature and precipitation in the five study areas during carnivore scat samplings (S1 Table), and general climatic categories of the five study areas (S2 Table). (DOCX)
