# Supplementary material for: Molecular validation of carnivore scat surveys: Effects of climate, scat age, and observer experience on identification success
Source: PLoS One. 2026 Feb 23;21(2):e0343095. doi: 10.1371/journal.pone.0343095 (PMC12928409; doi:10.1371/journal.pone.0343095)
Supplement: S1 File — (DOCX) [file pone.0343095.s001.docx]

**Supporting Information for:**

**Molecular validation of carnivore scat surveys: effects of climate, scat age, and observer experience on identification success**

Francisco Palomares^1*^, Jacinto Román^1^, Javier Calzada^2^, Juan Carlos Rivilla^1^, Irene Quintanilla^1^

^1^Department of Conservation Biology and Climate Change. Estación Biológica de Doñana-

CSIC. Av. Américo Vespucio 26, 41092-Sevilla, Spain

^2^Departamento de Ciencias Integradas y Centro de Estudios Avanzados en Física,

Matemáticas y Computación, Facultad de Ciencias Experimentales, Universidad de Huelva, 21071 Huelva, Spain

**S1 Table.** Mean temperature and precipitation for five protected areas in Spain (Aracena, Cazorla, Sierra Nevada, Montaña Palentina and Ordesa), calculated from January and February (winter) and from July and August (summer) for 2022 and 2023 according to AEMET data. Only seasons sampled in the area are shown.

| Study area | Year | Season | Mean Temp (°C) | Mean Precip (mm) |
| --- | --- | --- | --- | --- |
| Aracena | 2022 | Winter | 12.40 | 6.0 |
| Aracena | 2022 | Summer | 27.90 | 1.5 |
| Aracena | 2023 | Winter | 9.15 | 137.0 |
| Aracena | 2023 | Summer | 29.15 | 0.0 |
| Montaña Palentina | 2022 | Winter | 5.80 | 16.0 |
| Montaña Palentina | 2022 | Summer | 21.50 | 25.9 |
| Montaña Palentina | 2023 | Winter | 3.25 | 79.5 |
| Montaña Palentina | 2023 | Summer | 19.25 | 3.5 |
| Cazorla | 2022 | Winter | 10.65 | 18.2 |
| Cazorla | 2022 | Summer | 30.20 | 5.1 |
| Cazorla | 2023 | Winter | 8.75 | 23.5 |
| Cazorla | 2023 | Summer | 30.35 | 0.0 |
| Sierra Nevada | 2023 | Winter | 9.20 | 4.6 |
| Sierra Nevada | 2023 | Summer | 28.30 | 0.5 |
| Ordesa | 2023 | Summer | 16.45 | 57.6 |

**S2 Table.** Climatic categories of the five study areas in Spain (Aracena, Cazorla, Sierra Nevada, Montaña Palentina and Ordesa), obtained from the Iberian Climate Atlas (Agencia Estatal de Meteorología & Instituto de Meteorologia de Portugal, 2011).

| **Study Area** | **Main Category (Köppen)** | **Main Characteristics** |
| --- | --- | --- |
| Sierra Nevada | Dsb / Dsc | High-mountain cold climate with dry summers |
| Ordesa | Cfb / Dfb / ET | Transition from temperate conditions to tundra at higher elevations |
| Montaña Palentina | Cfb / Tipo D | Temperate climate without a dry season; cold conditions at high elevation |
| Cazorla | Csb | Temperate mountain climate with dry, mild summers |
| Aracena | Csa / Csb | Temperate Mediterranean climate influenced by altitude |

**References**

Agencia Estatal de Meteorología & Instituto de Meteorologia de Portugal. (2011). Atlas Climático Ibérico: Temperatura del aire y precipitación (1971-2000). Madrid: Ministerio de Medio Ambiente y Medio Rural y Marino. ISBN: 978-84-7837-079-5. DOI: 10.31978/784-11-002-5
